# Magnetic-Propelled Janus Yeast Cell Robots Functionalized with Metal-Organic Frameworks for Mycotoxin Decontamination

**DOI:** 10.3390/mi12070797

**Published:** 2021-07-05

**Authors:** Dongdong Lu, Songsong Tang, Yangyang Li, Zhaoqing Cong, Xueji Zhang, Song Wu

**Affiliations:** 1 Medical School, Anhui University of Science and Technology, Huainan 232001, China; ddlu@aust.edu.cn; 2 Institute of Urology, The Third Affiliated Hospital of Shenzhen University, Shenzhen 518000, China; liyangyang@zxbiomed.org (Y.L.); congzhaoqing@zxbiomed.org (Z.C.); 3 Shenzhen Following Precision Medical Research Institute, Luohu Hospital Group, Shenzhen 518000, China; 4 Research Center for Bioengineering and Sensing Technology, Beijing Key Laboratory for Bioengineering and Sensing Technology, Department of Chemistry & Biological Engineering, University of Science & Technology Beijing, Beijing 100083, China; zhangxueji@ustb.edu.cn

**Keywords:** yeast cell robots, Janus cell modification, magnetic propulsion, ZIF-67, mycotoxin decontamination

## Abstract

Cell robots that transform natural cells into active platforms hold great potential to enrich the biomedical prospects of artificial microrobots. Here, we present Janus yeast cell microrobots (JYC-robots) prepared by asymmetrically coating Fe_3_O_4_ nanoparticles (NPs) and subsequent in situ growth of zeolitic imidazolate framework-67 (ZIF-67) on the surface of yeast cells. The magnetic actuation relies on the Fe_3_O_4_ NPs wrapping. As the compositions of cell robots, the cell wall with abundant polysaccharide coupling with porous and oxidative ZIF-67 can concurrently remove mycotoxin (e.g., zearalenone (ZEN)). The magnetic propulsion accelerates the decontamination efficiency of JYC-robots against ZEN. Although yeast cells with fully coating of Fe_3_O_4_ NPs and ZIF-67 (FC-yeasts) show faster movement than JYC-robots, higher toxin-removal efficacy is observed for JYC-robots compared with that of FC-yeasts, reflecting the vital factor of the yeast cell wall in removing mycotoxin. Such design with Janus modification of magnetic NPs (MNPs) and entire coating of ZIF-67 generates active cell robot platform capable of fuel-free propulsion and enhanced detoxification, offering a new formation to develop cell-based robotics system for environmental remediation.

## 1. Introduction

Mycotoxin, a low molecular weight substance, is a secondary and toxic metabolite produced by fungi during regular life activities, such as Aflatoxins (AF), Ochratoxin A (OTA), T-2 toxin (T-2), and zearalenone (ZEN) [1,2,3]. These toxins are commonly produced in humid and dark conditions, especially where food and feed are stored [4]. When mycotoxin-contaminated entities enter the food chain, mycotoxins will gradually accumulate in living bodies, leading to mycotoxicosis and endangering the health of humans and animals [5]. For example, ZEN has estrogenic and anabolic abilities, and its accumulation in animals may cause hyperestrogenism and affect reproductive function [6]. Thus, developing a green and efficient method to remove mycotoxins and alleviate public concerns is urgently needed.

Biosorption is considered as a promising clean-up biotechnology, relying on the absorption of biological components [7]. For example, microorganisms (e.g., bacteria, fungi, yeast cells, and algae) and cells (e.g., macrophages, red blood cells, and platelets) have been used as biosorbents to remove organic and inorganic substances (e.g., heavy metals, toxins, and hormones) [8,9,10,11]. Among them, yeast cells, such as *Saccharomyces cerevisiae*, have an abundance of components on their surface, including polysaccharides, proteins, and lipids, endowing them with the superiority in absorbing pollutants through hydrogen bonds and ionic or hydrophobic interactions [2,5]. Moreover, *Saccharomyces cerevisiae* strains can be easily obtained in large quantity and are approved safely by the US Food and Drug Administration (FDA) and the European Food Safety Authority [12]. Such biocompatible biosorbents have exhibited advantages in removing mycotoxins [2,3,5,13,14,15,16,17]. However, the passive binding between cells and mycotoxin caused a time-consuming decontamination process and extended the period in potential threats. Therefore, leveraging biosorbents for rapid and active detoxification is highly demanded.

Microrobots have been emerging as the spotlight field benefiting from their autonomous movement and precise manipulation [18,19,20,21,22,23]. These tiny machines can convert and obtain propulsive force from surrounding energy, such as chemicals, light, ultrasound, and magnetic power [8,18,22,24,25,26]. Coupling microrobots with cells generates multifunctional cell robots with attractive performance in detoxification [10], cell manipulation [27,28,29,30,31], drug delivery [22,24], and tissue regeneration [32]. Building cell robots based on yeast cells holds great potential in realizing active biosorbents for accelerated environmental remediation. Considering the potential application with rigorous requirements of biocompatibility and motion reconfiguration, magnetic actuation is the prior choice to propel microrobots on account of its unique properties, such as fuel-free and salt-tolerance propulsion, remote and wireless control, and precise manipulation [8,24,25,33].

**Figure 1:**
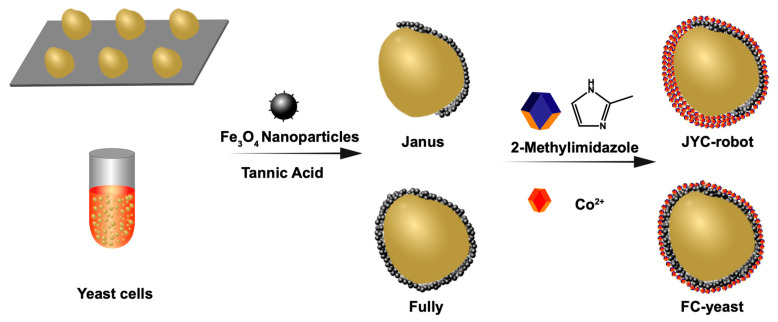
Schematic of the fabrication process of JYC-robots and FC-yeasts. For JYC-robots, yeast cells were first attached to the PLL-modified surface, followed with Janus modification of Fe_3_O_4_ NPs through TA complexation and in situ growth of ZIF-67 by mixing with precursors of cobalt nitrate and HmIm. For FC-yeasts, yeast cells in DIW were suspended in an Eppendorf tube, followed with fully coating of Fe_3_O_4_ NPs through TA complexation and in situ growth of ZIF-67.

In this study, we present magnetic-powered Janus yeast cell microrobots (JYC-robots) constructed by asymmetrically modifying Fe_3_O_4_ nanoparticles (NPs), followed with fully coating of zeolitic imidazolate framework-67 (ZIF-67) onto the surface of yeast cells (Figure 1). Artificial wrappings were verified with negligible impact on cell viability, demonstrating the biocompatibility of such cell surface engineering. Here, ZIF-67 is selected due to their porous structure and oxidative central ion of cobalt [34,35], representing attractive performance in environmental cleaning through absorption and oxidation [36]. JYC-robots exhibit effectively propulsion in various media under the magnetic field resulted from the magnet-responsive layer of Fe_3_O_4_ NPs. The biosorption of the cell wall and porous ZIF-67 can concurrently remove mycotoxin, which can be significantly enhanced by magnetic propulsion. More interestingly, we also fabricated yeast cells with fully coatings of Fe_3_O_4_ NPs and ZIF-67 (FC-yeasts). The entire encapsulation of Fe_3_O_4_ NPs leads to larger number of magnetic NPs (MNPs) on the cell surface, resulting in stronger response to external magnetic field and inducing faster motion than JYC-robots. However, magnetic-propelled JYC-robots still show higher efficacy in removing ZEN than that of FC-yeasts. Such difference is a result of the larger exposed surface of JYC-robots, revealing the superior function of the cell wall in binding with mycotoxin. The Janus modification is preferable in designing yeast cell robots with fuel-free propulsion for accelerating mycotoxin removal. Coupling MNPs and metal-organic frameworks (MOF) with yeast cells generates active biogenic magnetic catalysts, holding considerable potential in designing versatile cell robots for environmental remediation.

## 2. Materials and Methods

### 2.1. Fabrication of JYC-Robots

Yeast cells were cultured in a 15-mL Eppendorf tube with Yeast Extract Peptone Dextrose Medium (YPD) upon gentle shaking at 170 rpm at 28 °C. The YPD was prepared by mixing 20 g of peptone and 10 g of yeast extract in 900 mL of deionized water (DIW) with autoclavation, followed with adding 100 mL of sterilized 20% dextrose solution. Yeast cells were collected by centrifugation (4000 rpm, 3 min) and washed with DIW twice, then resuspended in DIW at room temperature (RT) until use. For poly(L-lysine) (PLL), a modified 12-well plate, 250 μL of PLL (MilliporeSigma, Burlington, NJ, USA) solution was added to the well and the plate was put in the oven at 60 °C for drying. Subsequently, yeast cells (1.2 × 10^7^) were added to the well and centrifuged (2000 rpm, 3 min) to attach them with the PLL-modified bottom surface. After removing the suspension and washing with DIW, 988 μL of DIW, 2 μL of Fe_3_O_4_ NPs solution (0.25 g/mL, Macklin, Shanghai, China), and 10 μL of tannic acid (TA) solution (4 mg/mL, MilliporeSigma, Burlington, NJ, USA) were added to the well plate and incubated for 3 min with gently shaking. The resulting yeast cells with Janus coating of Fe_3_O_4_ NPs (Fe_3_O_4_-J@Yeasts) were released from the PLL-modified surface with repeated and mild pipetting, followed with two DIW washes and suspension in 820 μL of DIW. Then, 100 μL of cobalt nitrate aqueous solution (11.64 mg/mL, Aladdin, Shanghai, China) and 80 μL of 2-methylimidazole (HmIm) aqueous solution (16.42 mg/mL, MilliporeSigma, Burlington, NJ, USA) were mixed with Fe_3_O_4_-J@Yeasts solution and incubated for 10 min under mild shaking. Last, the fabricated JYC-robots were collected and dispersed in DIW at RT until use after DIW wash.

### 2.2. Fabrication of FC-Yeasts

The fully coating of Fe_3_O_4_ NPs was carried out in 1.5-mL Eppendorf tubes. Yeast cells were functionalized with the same reagents at the same concentration as the preparation process of JYC-robots. The resulting FC-yeasts were suspended in DIW and stored at RT for use.

### 2.3. Characterization of Yeast Cell Robots

Fe_3_O_4_ NPs and ZIF-67 were labeled with fluorescein isothiocyanate (FITC) and Rhodamine B (RhB), respectively, for fluorescent characterization. For FITC-labeled Fe_3_O_4_ NPs, 2 μL of Fe_3_O_4_ NPs solution was dispersed in 998 μL of dopamine-HCl (DA, Aladdin, Shanghai, China) solution (2 mg/mL, phosphate-buffered saline (PBS), pH = 8.5) with overnight stirring [37]. The resulting polydopamine-coated Fe_3_O_4_ (PDA@Fe_3_O_4_) NPs were separated by magnet and washed twice with DIW, and then resuspended in 400 μL of PBS (pH = 8.5) at 4 °C until use. The PDA structure is known to have abundant chemical groups, such as the amino group [38], enabling reaction with 5(6)-carboxyfluorescein N-hydroxysuccinimide ester (NHS-FITC, Macklin, Shanghai, China). Then, 0.4 mg of NHS-FITC was dissolved in a mixture of 100 μL of DMSO and 300 μL of DIW, and then mixed with 400 μL of PDA@Fe_3_O_4_ solution. After shaking for 1 h, FITC-labeled PDA@Fe_3_O_4_ was collected with magnet and wash three times with DIW. The final products were suspended in 100 μL of PBS (pH = 8.5) at 4 °C until use. To label ZIF-67 with RhB, 7 μL of saturated RhB (Macklin, Shanghai, China) solution was added during the in situ growth of ZIF-67. RhB can be encapsulated inside ZIF-67 through the biomineralization process [39]. The resulting samples were collected by centrifugation and washed with DIW three times to remove the free dyes. Fluorescent images were captured with a laser confocal microscope (ZEISS LSM 800) with a 63× oil objective. Flow cytometry was applied to analyze the wrapping efficiency of ZIF-67 using a BD FACSCalibur flow cytometer.

The cell morphology was characterized by scanning electron microscope (SEM) and energy-dispersive X-ray spectroscopy (EDX) images taken by Thermo APREO S instrument (Thermo Fisher Scientific, Waltham, MA, USA).

### 2.4. Cell Viability Evaluation

Fluorescein diacetate (FDA, Macklin, Shanghai, China) assay was performed to assess cell viability after Fe_3_O_4_ NPs and ZIF-67 coating. Fabricated cell robots were incubated with the FDA solution (50 μg/mL) for 20 min at 30 °C, followed with three times DIW wash to remove the free dyes. Fluorescent images were taken by ZEISS LSM 800 with a 63× oil objective.

### 2.5. Motion Study

All of the experiments were carried out in DIW except the exploration in various media. The motion videos were captured using an inverted optical microscope (MshOt) with a 45× objective and a NI LabVIEW software was used to control the rotating magnetic field (RMF). The motion analysis was conducted in NIS-Elements AR 5.2 software.

### 2.6. ZEN Decontamination

The ZEN solution was prepared by dissolving 12 μg of ZEN in 120 μL of DIW and incubated with various conditions, including uncoated yeast cells, yeast cells with full coatings of Fe_3_O_4_ NPs (Fe_3_O_4_-F@Yeasts), yeast cells with full coatings of Fe_3_O_4_ NPs (Fe_3_O_4_-J@Yeasts), heat-killed yeast cells with Janus coating of Fe_3_O_4_ NPs (Fe_3_O_4_-J@killed-Yeasts), yeast cells with ZIF-67 coating (ZIF-67@yeasts), FC-yeasts, FC-yeasts under the RMF, JYC-robots, and JYC-robots under the RMF. The cell concentration in each group is the same at 1.5 × 10^5^. After incubation, the resulting mixture was centrifuged at 4000 rpm for 3 min and the supernatant was collected to quantify the remaining ZEN by fluorescence detection using a multifunctional microplate detector (Spark, Tecan, Männedorf, Switzerland). For heat-killed yeast cells, yeast cell suspension was bathed in water at 100 °C for 30 min, then heat-killed yeast cells were collected and dispersed in DIW at RT until use after DIW wash.

## 3. Results and Discussion

### 3.1. Fabrication and Characterization of JYC-Robots and FC-Yeasts

**Figure 2:**
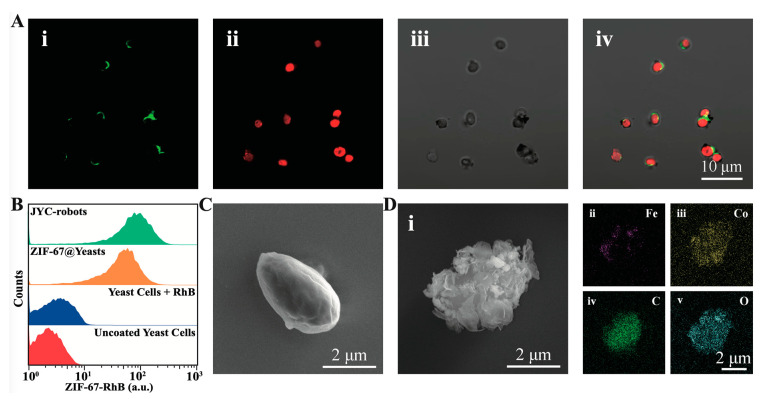
Characterization of JYC-robots. (**A**) CLSM images of JYC-robots: i: FITC channel; ii: RhB channel; iii: bright-field channel, iv: merged image of FITC, RhB, and bright-field channels. (**B**) Representative flow cytometry histograms of JYC-robots (cyan), ZIF-67@Yeasts (orange), yeast cells mixed with RhB (blue), and uncoated yeast cells (red). (**C**) SEM image of uncoated yeast cells. (**D**) SEM and corresponding EDX images of the JYC-robot.

The fabrication process of JYC-robots is illustrated in Figure 1 (see details in experimental section). The Janus modification of Fe_3_O_4_ NPs on the surface of yeast cells was performed according to the method described in a previous study [22]. Briefly, yeast cells were attached to the PLL-modified surface in a commercial 12-well plate for partially blocking the cell surface. Then, Fe_3_O_4_ NPs and TA solution were added and reacted for 3 min. The phenolic hydroxyl groups in TA’s structure complex metals and compound [40,41,42,43,44,45], leading to the Janus wrapping of MNPs on the surface of yeast cells. The resulted products (Fe_3_O_4_-J@Yeasts) were dissociated from the PLL surface and transferred to an Eppendorf tube for the following in situ MOF encapsulation. The Fe_3_O_4_-J@Yeasts suspension was mixed with cobalt nitrate hexahydrate and then the aqueous solution of HmIm was dropwise added along with gentle shaking for 10 min. The fabricated JYC-robots were collected via centrifugation and washed with DIW to remove excess MOF precursor. To demonstrate the successful fabrication of JYC-robots, FITC (excitation/emission = 488/525 nm) and RhB (excitation/emission = 540/625 nm) were used to label Fe_3_O_4_ NPs and ZIF-67, respectively, for fluorescent characterization. The confocal laser scanning microscopy (CLSM) images are shown in Figure 2A. The FITC channel (i) illustrates the crescent shape of FITC-labeled Fe_3_O_4_ NPs, whereas RhB fluorescence (ii) is uniformly distributed on the cell surface. The merged image (iv) shows the co-localization of RhB and FITC signals on yeast cells. Such results confirm the asymmetric modification of Fe_3_O_4_ NPs and fully coating of ZIF-67 on the yeast cells surface. Moreover, yeast cells still maintain their intrinsically ellipsoidal shape after cell surface modification. Flow cytometry was applied to explore the coating efficiency of ZIF-67. As shown in Figure 2B, strong RhB signal (~92%) was observed for yeast cells with ZIF-67 coating and JYC-robots, whereas control experiments containing uncoated yeast cells and yeast cells mixed with RhB did not show the RhB signal. Such results further verify the feasibility of subsequent ZIF-67 growth on Fe_3_O_4_-J@Yeasts to form JYC-robots.

The surface morphology of fabricated JYC-robots was examined by SEM images. Compared to the smooth surface of unmodified yeast cell (Figure 2C), the rough and fold surface of the JYC-robot reveals the successful encapsulation of MNPs and ZIF-67 (Figure 2D, (i). EDX images illustrate the presence and elemental distribution of Fe (ii), Co (iii), C (iv), and O (v) on the cell surface. The uneven distribution of Fe and homogeneous cover of Co are consistent with the fluorescent results shown in Figure 2A. SEM and EDX images of multiple JYC-robots are shown in Figure S1. Furthermore, we explored the effects of such cell surface engineering on cell viability through FDA assay [46], where live cells can uptake FDA and decompose them to fluoresce. The strong green fluorescence shown in Figure S2 validates the living state of JYC-robots, reflecting the biocompatibility of artificial wrappings without fixing yeast cells.

Here we also fabricated FC-yeasts with entire modification of Fe_3_O_4_ NPs and ZIF-67 to compare the effect of coating methods (Janus or fully) on magnetic propulsion. The preparation process of FC-yeasts is displayed in Figure 1 (see details in experimental section), which was proceeded in the Eppendorf tube with the same procedures as the aforementioned descriptions, where the input concentrations of Fe_3_O_4_ and precursors of ZIF-67 were oversaturated to achieve maximum loading of MNPs and ZIF-67 on the yeast cell surface. Figure S3A displays the CLSM images of FC-yeasts, revealing the uniform distribution of FITC-labeled Fe_3_O_4_ NPs and RhB-labeled ZIF-67 on the cell surface. SEM (i) and EDX (ii–v) images in Figure S3B and Figure S4 show bumpy morphology of FC-yeasts and homogeneous coverage of Fe and Co elements, respectively, in accordance with the fluorescent images in Figure S3A. Such fully coating on cell surface also did not alter the cell viability, validated by the FDA fluorescence presented in Figure S3C.

Overall, these results demonstrate the successful fabrication of JYC-robots with Janus coating of Fe_3_O_4_ NPs and entire coverage of ZIF-67, and FC-yeasts with entire wrapping of Fe_3_O_4_ NPs and ZIF-67, allowing biomedical applications by leveraging functionalities of cells and artificial coatings.

### 3.2. Motion Performance of JYC-Robots and FC-Yeasts

**Figure 3:**
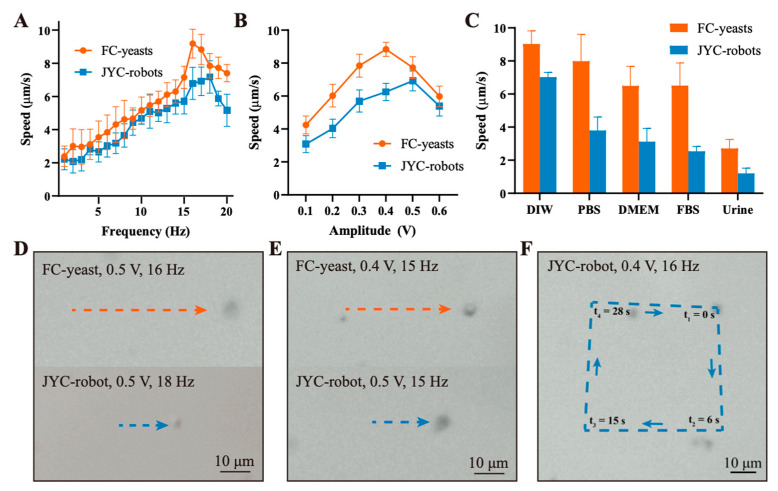
Propulsion performance of JYC-robots and FC-yeasts. Speed comparison of JYC-robots and FC-yeasts under (**A**) various frequencies (amplitude: 0.5 V) and (**B**) various amplitudes (frequency: 15 Hz). (**C**) Motion speed of JYC-robots and FC-yeasts in various media under magnetic field (0.4 V, 16 Hz). (**D**) Optical tracking trajectories over 5 s of JYC-robots and FC-yeasts at a constant amplitude of 0.5 V, taken from Movie S1. (**E**) Optical tracking trajectories over 5 s of JYC-robots and FC-yeasts at constant frequency of 15 Hz, taken from Movie S2. (**F**) Optical tracking trajectories of JYC-robots under magnetic control with a predefined path of square (□)-like shape (RMF: 0.4 V and 16 Hz, taken from Movie S5).

**Figure 4:**
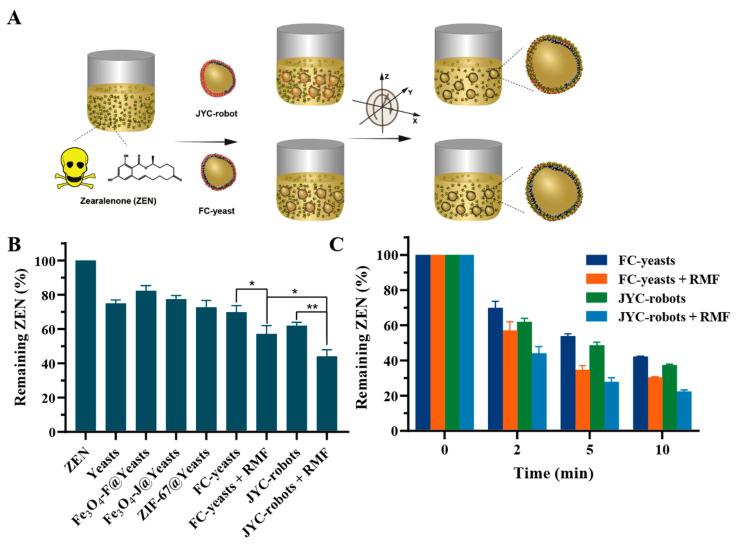
ZEN decontamination with JYC-robots and FC-yeasts. (**A**) Schematic of JYC-robots and FC-yeasts for active ZEN decontamination under the RMF. (**B**) ZEN removal after incubated with various conditions for 2 min (RMF: 0.4 V and 16 Hz). *n* = 3, mean ± SD. * *p* < 0.05, ** *p* < 0.01. (**C**) Evaluation of the decontamination performance of JYC-robots and FC-yeasts without or with the RMF (0.4 V and 16 Hz) at different time-points.

The successful encapsulation of superparamagnetic Fe_3_O_4_ NPs on cell surface bestows such cell robots with attractive motion performance under the external RMF [8]. Figure 3A shows the motion speed of JYC-robots and FC-yeasts under different frequencies at constant amplitude of 0.5 V. As the frequency increases, the velocities of both types of cells first increase and reach the maximum average speed of 7.2 μm/s at 18 Hz and 9.2 μm/s at 16 Hz for JYC-robots and FC-yeasts, respectively, followed with a speed decrease due to the step-out frequency effects [24]. Corresponding optical trajectories of both cells at maximum speed are illustrated in Figure 3D (Movie S1). Yeast cell robots are observed moving along the minor axis of their ellipsoidal shape under the magnetic field. A similar trend is observed in Figure 4B, exhibiting the relationship between motion speed and the amplitude of the RMF at the constant frequency of 15 Hz. The maximum speed of 6.9 μm/s at 0.5 V and 8.8 μm/s at 0.4 V are achieved for JYC-robots and FC-yeasts, respectively. Figure 3E displays representative motion trajectories for two types of cells at their peak speed under 15 Hz (Movie S2). It is observed that JYC-robots exhibit lower speed than FC-yeasts. Such difference can be attributed to the amount of MNPs on the cell surface, directly affecting the magnetic response to external RMF. FC-yeasts with completed encapsulation of Fe_3_O_4_ NPs have more MNPs compared with JYC-robots, leading to faster motion under magnetic control. Considering applying such cell robots in potentially biomedical fields, we evaluated their magnetic propulsion in biological fluids. As shown in Figure 3C, at the RMF with an amplitude of 0.4 V and frequency of 16 Hz, JYC-robots and FC-yeasts show effective propulsion in DIW, PBS, Dulbecco’s modified Eagle’s medium (DMEM), fetal bovine serum (FBS), and urine. The speed decreases from DIW to urine may ascribe to the local viscosity of each environment. The corresponding propulsion trajectories are displayed in Figure S5 (Movie S3) and Figure S6 (Movie S4). Furthermore, the precise control of the magnetic field allows us to navigate JYC-robots under a predefined path. As shown in Figure 3F, the JYC-robot undergoes a square-like (□) trajectory upon a programmed magnetic field (Movie S5). Overall, these results demonstrate that JYC-robots exhibit effective magnetic propulsion in various biological media, enabling the integration of yeast cells with asymmetric coating of MNPs and full coating of MOF into microrobotic community and expanding the biohybrid design of artificial microrobots for versatile applications. The propulsion performance is related to the amount of Fe_3_O_4_ NPs, where the movement of FC-yeasts is faster than JYC-robots. The capability to move along a predesigned loop offers the possibility to precisely navigate magnetic-powered yeast cell robots under complex environments.

### 3.3. Enhanced ZEN Removal Using JYC-Robots and FC-Yeasts

The motile JYC-robots and FC-yeasts allow us to investigate their detoxification performance under the RMF (Figure 4A). Here, ZEN was selected as the model mycotoxin. Figure 4B shows the remaining ZEN after incubated with various conditions for 2 min. The groups of yeasts, Fe_3_O_4_-F@Yeasts, Fe_3_O_4_-J@Yeasts and ZIF-67@Yeasts were acted as negative controls. After incubated with ZEN solution, yeasts absorbed 25% of ZEN. Fe_3_O_4_-F@Yeasts removed 18% of ZEN. Comparable efficiency around 25% was obtained after incubated with Fe_3_O_4_-J@Yeasts and ZIF-67@Yeasts. Such results reveal the capability of yeast cell walls and ZIF-67 to remove mycotoxin. The abundant polysaccharides (85–90%) in yeast cell walls is responsible for binding ZEN. Meanwhile, the ZIF-67 wrapping with porous structure and active oxidation of cobalt ion [34,35] can remove ZEN by adsorption and oxidization. The static FC-yeasts and JYC-robots bind 30% and 38% of ZEN, respectively. In contrast, when external RMF was applied, FC-yeasts and JYC-robots achieved significant enhancement in ZEN removal efficacy at 43% and 56%, respectively. Such results indicate that the magnetic propulsion actuated by RMF can accelerate the binding between cell robots and ZEN. The rapid movement of cell robots can enhance mass transport in ZEN solution [47], further improving the detoxification efficacy. Mechanical shaking also brings mixing and microconvection of the solution. As shown in Figure S9, JYC-robots under shaking exhibited lower detoxification efficacy (45%) compared to that of JYC-robots under RMF. Such results may be attributed to the small aggregates of JYC-robots formed under RMF and more effective local mixing caused by effective magnetic propulsion of JYC-robots [8,48,49]. We also explored the effect of yeast concentrations on the removal efficacy of ZEN. As shown in Figure S10, the removal efficacy increased with the rise of yeast concentrations, and reached 52% when the yeast concentration was 1.2 × 10^6^_,_ which was comparable to that of JYC-robots under magnetic field. Although simply increasing the concentration of yeast cells can enhance the detoxification efficacy, abundant residual yeast cells may cause concern as they are hard to separate from the purified solution, causing secondary pollution and potential health problems when they entered the food chain. Yeast cell robots with simple and large-scale fabrication can significantly improve the removal efficacy of mycotoxin under RMF, decreasing the dosage of biosorbents and reducing unwanted side effects. It is noteworthy that motile JYC-robots exhibit more efficient decontamination than that of FC-yeasts. As the outer layers of both cells are porous ZIF-67, mycotoxins can penetrate the porous cavity of ZIF-67 and bind with the cell wall. The larger unmasked cell surface of JYC-robots leads to higher removal efficiency of ZEN. Yeast cells treated at 100 °C for 30 min were asymmetrically coated with MNPs (Fe_3_O_4_-J@killed-Yeasts) to perform ZEN removal, where cells were already killed and verified by FDA staining (Figure S7). The ZEN removal efficiency of Fe_3_O_4_-J@killed-Yeasts was comparable with that of Fe_3_O_4_-J@Yeasts (Figure S8). Short-term treatment at high temperature has a small impact on polysaccharide structure [50]. Such results highlight that mycotoxin removal is determined by polysaccharides on yeast cell walls and independent to yeast cell viability. Figure 4C exhibits the time-dependent toxin-removal under various conditions at different time points. The general trend is that the removal efficiency of ZEN increases along with the raise of time duration. At each time point, the active cell platforms present enhanced toxin elimination than static counterparts and JYC-robots still show superior performance in ZEN-removal in contrast with FC-yeasts. In the starting 5 min, magnetic-propelled yeast cells can rapidly absorb ZEN and the remaining ZEN is 35% for FC-yeasts and 28% for JYC-robots, respectively. However, the removal efficacy tends to be saturated at 10 min and may be a result of the limited cell robots with confined site to bind with mycotoxin. Overall, these results demonstrate that yeast cells and ZIF-67, the components of cell robots, can concurrently remove ZEN. The magnetic propulsion of cell robots can accelerate their binding with mycotoxin and improve detoxification efficiency. JYC-robots represent enhanced removal efficacy of ZEN compared to FC-yeasts due to the larger exposed cell surface.

## 4. Conclusions

In conclusion, we developed a JYC-robot by asymmetrically modifying Fe_3_O_4_ NPs and then in situ growing ZIF-67 on the surface of yeast cells. The cells after inorganic coating are verified in live status. The MNPs’ modification bestows JYC-robots with efficiently magnetic propulsion in various media. The strong absorption performance and the oxidative cobalt ions of ZIF-67 act as the synergistical factor with yeast cells in mycotoxin removal. The effective movement of JYC-robots under magnetic field can accelerate the absorption and binding of ZEN, further enhancing the detoxification efficacy compared to the passive counterparts. Interestingly, FC-yeasts were also constructed and exhibit faster movement in contrast with JYC-robots. However, more efficient detoxification was observed when incubated with magnetic-propelled JYC-robots compared to that of FC-yeasts due to the larger exposed cell surface, revealing the significant impact of cell wall on absorbing mycotoxin. JYC-robots benefits from the Janus modification to simultaneously achieve fuel-free propulsion and impressive performance in detoxification by leveraging the cell function and MOF wrappings. Coating yeast cells with MNPs and MOF yields biogenic magnetic catalysts with self-propulsion and enhanced capability in environmental remediation, such as mycotoxin removal in drinking water sources, and can be readily expanded to other types of cells and artificial wrappings in designing versatile cell robots for broader applications, such as drug delivery, biosensing, and bioconversion [45,51,52]. Future efforts may lead to simplify fabrication process with one-step method to synthesize magnetic ZIF composites [53] and develop biocompatible coatings, such as ZIF-8 [54]. Such magnetic-powered microsystem may also face constrains as large and complex requirements may be required in practical applications, causing money-consuming and inconvenient process. Besides, the limited chamber of such devices hinders the application of magnetic-propelled yeast cell robots in large-scale decontamination. The presented platform can be potentially applied for the demand of a small amount, such as detoxification of ethanol fermentation and poultry feed. Despite these limitations, the superiorities of magnetic field with remote and precise control can realize rapid separation of biosorbents and arrival of hard-to-reach site, avoiding secondary pollution caused by residual agents and broadening the operation scope of such decontamination process in complex and dynamic environments. However, the magnetically separated cell robots are not allowed to be reused, because, to the best of our knowledge, the binding between mycotoxin and yeast cells or ZIF-67 cannot be reversed.

## Supplementary Materials

The following are available online at https://www.mdpi.com/article/10.3390/mi12070797/s1, Figure S1: SEM and EDX images of two JYC-robots, Figure S2: CLSM images of JYC-robots with FDA staining, Figure S3: Characterization of FC-yeasts, Figure S4: SEM and EDX images of multiple FC-yeasts, Figure S5: Motion trajectories over 5 s of JYC-robots in various media under RMF (0.4 V, 16 Hz, taken from Movie S3), Figure S6: Motion trajectories over 5 s of FC-yeasts in various media under RMF (0.4 V, 16 Hz, taken from Movie S4), Figure S7: CLSM images of yeast cells and heat-killed yeast cells with FDA staining, Figure S8: ZEN removal after incubated with Fe3O4-J@Yeasts and Fe3O4-J@killed-Yeasts for 2 min, Figure S9: ZEN removal after incubated with JYC-robots under RMF (JYC-robots + RMF) or shaking (JYC-robots + shaking) for 2 min, Figure S10: ZEN removal after incubated with yeast cells upon different concentrations for 2 min, Movie S1: Propulsion performance of the JYC-robot and FC-yeast under a rotating magnetic field at the amplitude of 0.5 V, Movie S2: Propulsion performance of the JYC-robot and FC-yeast under a rotating magnetic field at the frequency of 15 Hz, Movie S3: Propulsion performance of JYC-robots in various media under a rotating magnetic field (0.4 V, 16 Hz), Movie S4: Propulsion performance of FC-yeasts in various media under a rotating magnetic field (0.4 V, 16 Hz), Movie S5: Motion trajectory of a JYC-robot under the rotating magnetic field with a predefined path of square-like shape (□) (RMF: 0.4 V and 16 Hz).
