# Supplementary material for: Magnetic-Propelled Janus Yeast Cell Robots Functionalized with Metal-Organic Frameworks for Mycotoxin Decontamination
Source: Micromachines (Basel). 2021 Jul 5;12(7):797. doi: 10.3390/mi12070797 (PMC8307641; doi:10.3390/mi12070797)
Supplement: Supplementary file 1 [file micromachines-12-00797-s001.zip › Supporting.pdf]

Supporting Information

Magnetic-propelled Janus yeast cell robots functionalized with metal-organic frameworks for mycotoxin decontamination

Dongdong Lu [1,3,4], Songsong Tang [2,*], Yangyang Li [3], Zhaoqing Cong [3], Xueji Zhang [2], and Song Wu [1,3,4,*]

Movie S1: Propulsion performance of the JYC-robot and FC-yeast under a rotating magnetic field at the amplitude of 0.5 V.

Movie S2: Propulsion performance of the JYC-robot and FC-yeast under a rotating magnetic field at the frequency of 15 Hz.

Movie S3: Propulsion performance of JYC-robots in various media under a rotating magnetic field (0.4 V, 16 Hz).

Movie S4: Propulsion performance of FC-yeasts in various media under a rotating magnetic field (0.4 V, 16 Hz).

Movie S5: Motion trajectory of a JYC-robot under the rotating magnetic field with a predefined path of square-like shape (□) (RMF: 0.4V and 16 Hz).

Supporting Figures:

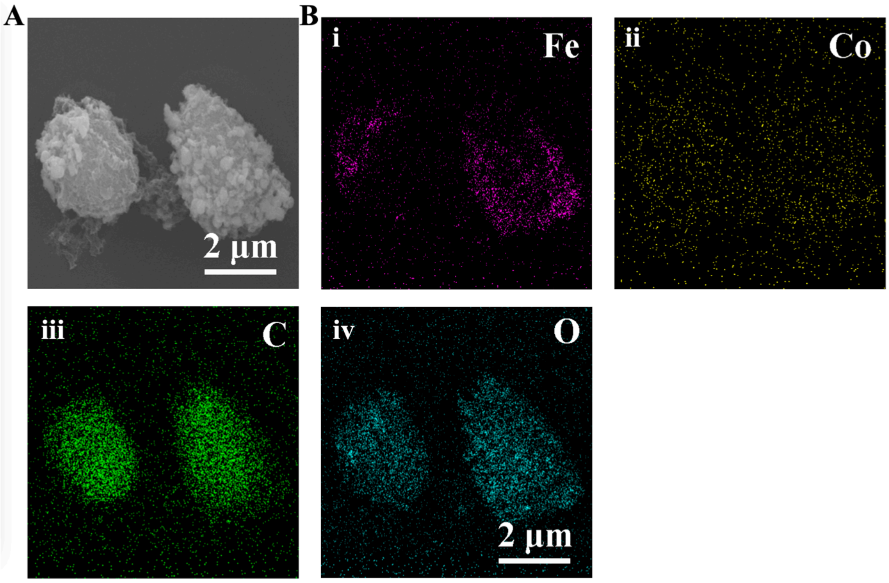

Figure S1. SEM and EDX images of two JYC-robots.

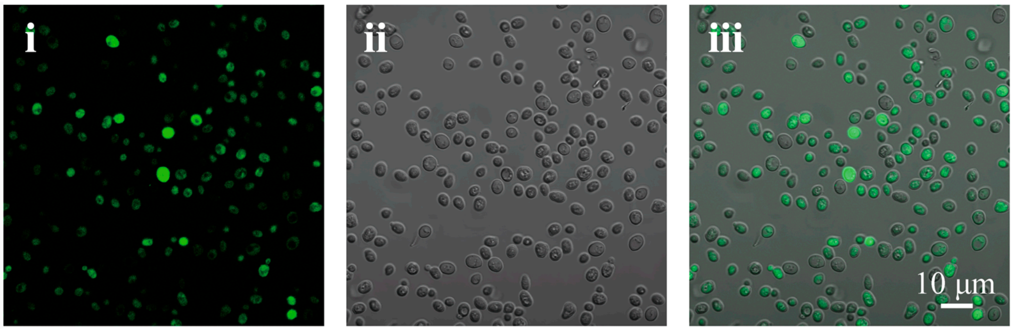

Figure S2. CLSM images of JYC-robots with FDA staining: i: FDA channel; ii: bright-field channel, iii: merged image of FDA and bright-field channels.

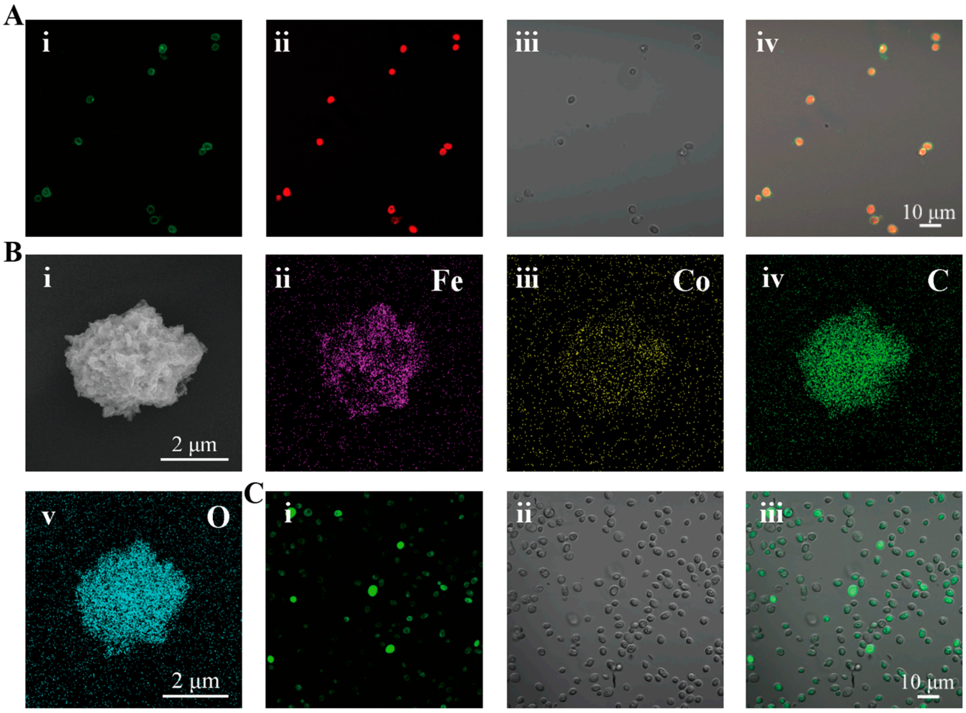

Figure S3. Characterization of FC-yeasts. (A) CLSM images of FC-yeasts: i: FITC channel; ii: RhB channel; iii: bright-field; iv: merged image of FITC, RhB, and bright-field channels. (B) SEM and corresponding EDX images of the FC-yeast. (C) CLSM images of FC-yeasts with FDA staining: i: FDA channel; ii: bright-field; iii: merged image of FDA and bright-field channels.

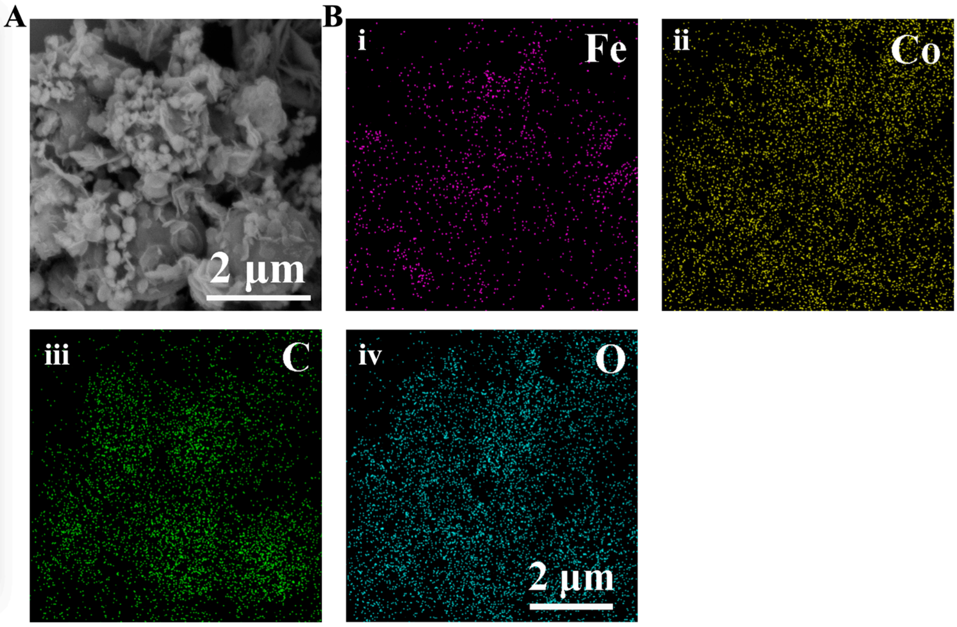

Figure S4. SEM and EDX images of multiple FC-yeasts.

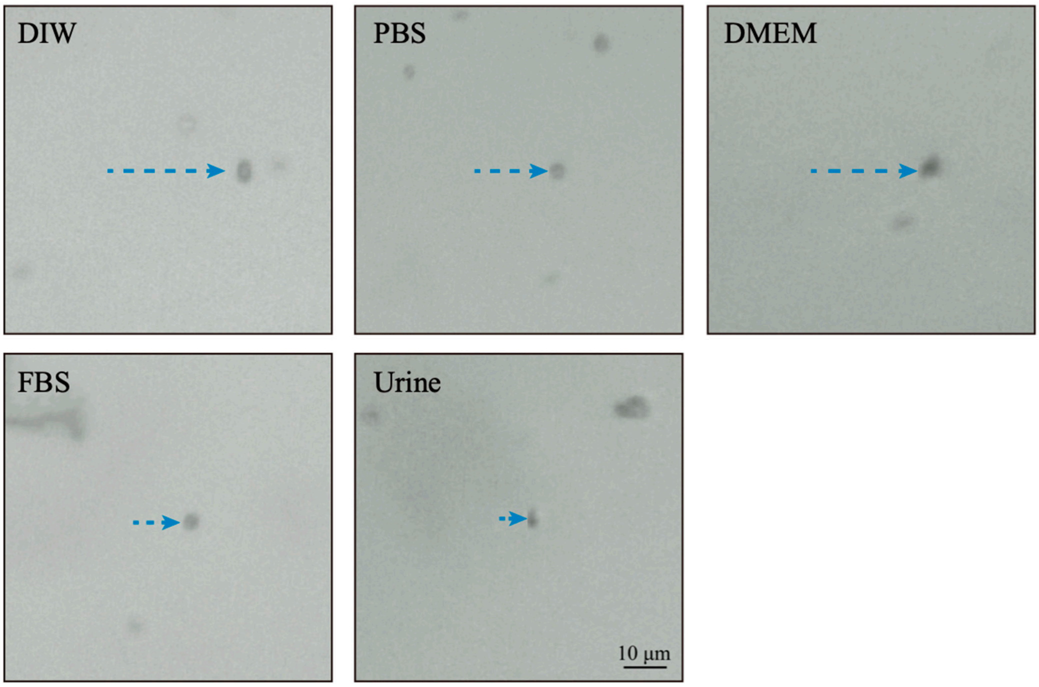

Figure S5. Motion trajectories over 5 s of JYC-robots in various media under the RMF (0.4 V, 16 Hz, taken from movie S3).

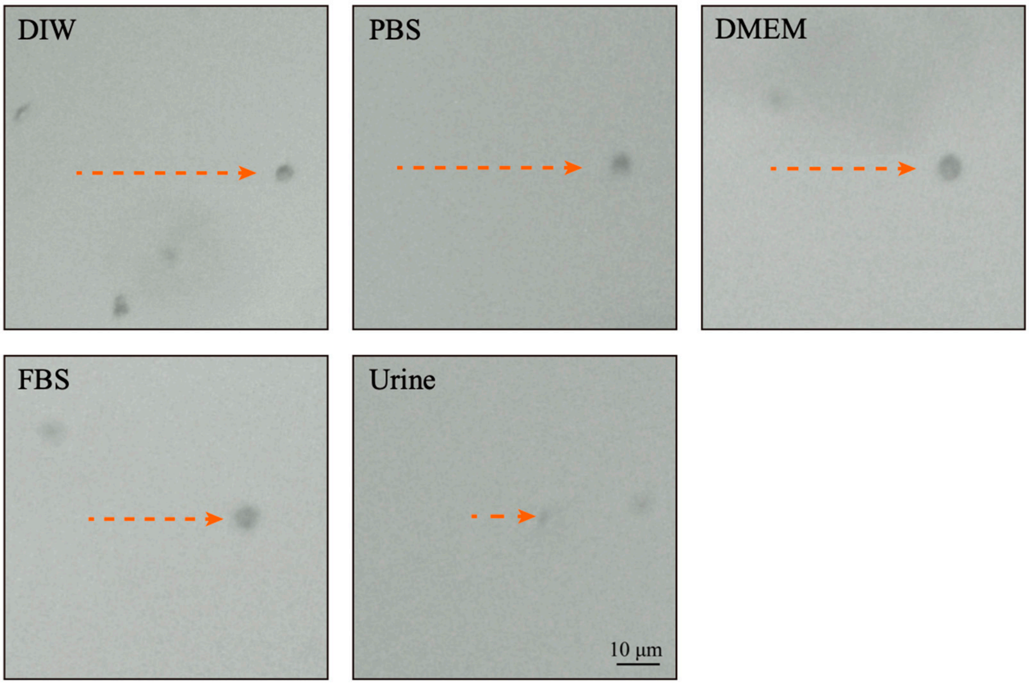

Figure S6. Motion trajectories over 5 s of FC-yeasts in various media under the RMF (0.4 V, 16 Hz, taken from movie S4).

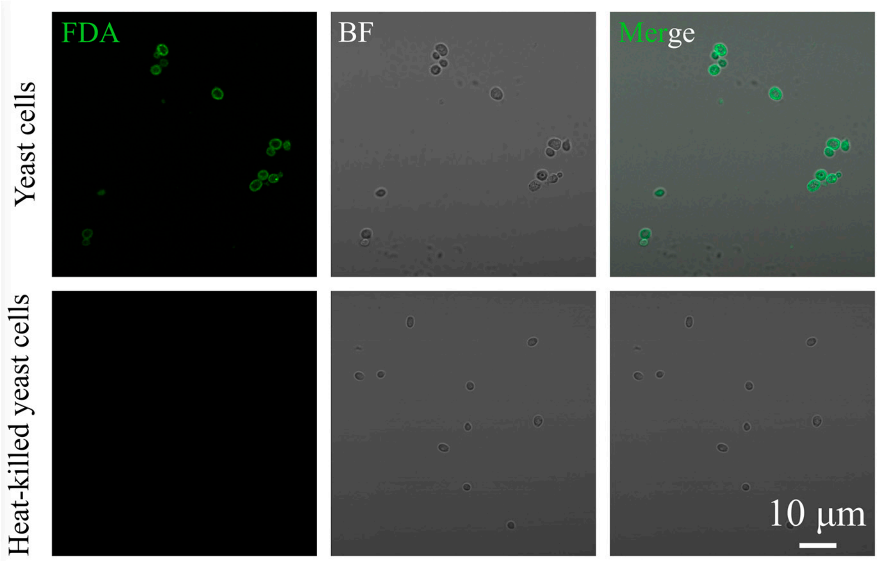

Figure S7. CLSM images of yeast cells and heat-killed yeast cells with FDA staining. BF: bright-field.

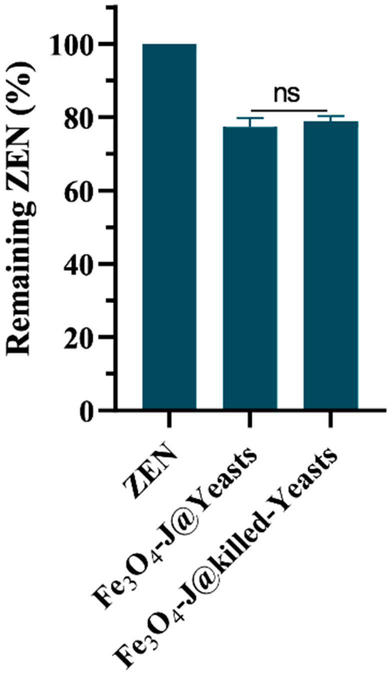

Figure S8. ZEN removal after incubated with Fe_3O_4-J@Yeasts and Fe_3O_4-J@killed-Yeasts for 2 min. n=3, mean ± SD.

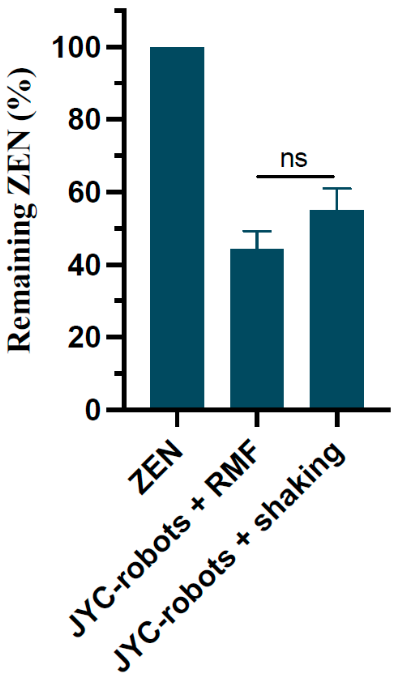

Figure S9. ZEN removal after incubated with JYC-robots under RMF (JYC-robots + RMF) or shaking (JYC-robots + shaking) for 2 min. n=3, mean ± SD.

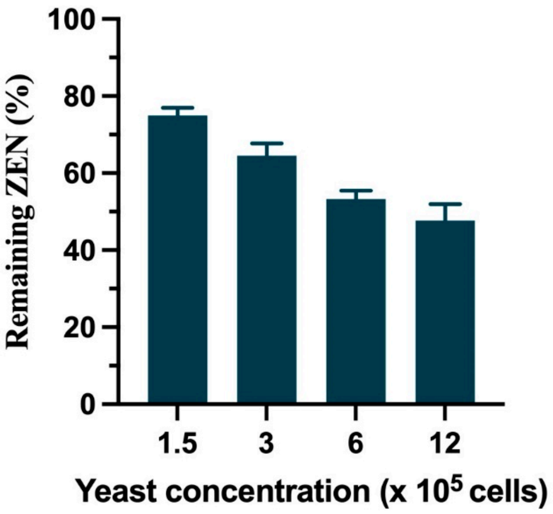

Figure S10. ZEN removal after incubated with yeast cells upon different concentrations for 2 min. n=3, mean ± SD.
